# Cross-Cultural Adaptation and Validation of the Korean Version of the Dundee Ready Education Environment Measure (DREEM)

**DOI:** 10.1155/2021/5591911

**Published:** 2021-08-13

**Authors:** Hyunho Kim, Pyeongjin Jeon, Seungju Kim, Jiseong Hong, Yeonseok Kang

**Affiliations:** ^1^7 Days Inc., Seoul, Republic of Korea; ^2^Boneline Korean Medicine Clinic, Seoul, Republic of Korea; ^3^Sarangin Korean Medicine Clinic, Ansan, Republic of Korea; ^4^Research Center of Korean Traditional Medicine, Wonkwang University, Iksan, Republic of Korea; ^5^Department of Medical History, College of Korean Medicine, Wonkwang University, Iksan, Republic of Korea; ^6^Institute of Korean Medicine Education and Evaluation, Seoul, Republic of Korea

## Abstract

Internationally, Dundee Ready Education Environment Measure (DREEM) is being used to evaluate and compare students' awareness regarding medical education environment. This study aimed to adapt DREEM into Korean, to evaluate the reliability and validity, and to compare its structure to the original DREEM structure. The DREEM was translated using 6 steps which were suggested in cross-cultural adaptation protocols: translation, synthesis, back translation, expert committee review, pilot test, and psychometric study (*N* = 451). We performed confirmatory factor analysis including basic analysis. For evaluating the original model's goodness of fit with the acquired dataset, model fit indices and construct validity were discussed. The Korean version was completed upon through cross-cultural adaptation protocols. Statistical analysis with 451 data sets showed that the root mean square error of approximation = 0.06, goodness-of-fit index = 0.75, and Tucker–Lewis index = 0.73. Almost construct reliabilities were all over 0.707. Except for just one pair, all squares of correlation coefficients were greater than the corresponding average variance extracted. In conclusion, we developed the Korean version of DREEM. Although the original 5-factor structure was acceptable, low convergent and discriminant validity indices suggested that further studies for the Korean environment are necessary for the respecified or modified factor structures.

## 1. Introduction

Medical education's principal aim is to produce medical doctors who will be ready to serve the fundamental purposes of medicine: curing disease and participating in all social aspects related to medicine and healthcare [1]. Therefore, many countries have tried to build effective and meaningful medical education systems. Furthermore, educator-centered and nonintegrated education systems have been transformed into student-centered and integrated learning systems [2–7]. Medical education is composed of several factors, of which educational environment is one of the most important [8]. Educational environment is a very complex area that, in a comprehensive sense, encompasses the physical location, cultural curriculum, educational facilities, and methods of education [4, 9]. Educational environments are as important as expertise delivery in medical education [8]. Educational environments contribute to students' achievements, satisfaction, healthy competition, independence, self-confidence, critical thinking, aspirations, and so on [4, 10–14]. Accordingly, it is very important to provide an appropriate environment for facilitating effective medical education [15].

Therefore, educators and policy makers should consider the educational environments in order to improve education quality, and researchers have developed various tools for evaluating educational environments [9, 16, 17]. Pace and Stern developed the College Characteristics Index (CCI) to understand students' perceptions of educational environment [18]. Hutchins developed the Medical School Environment Inventory (MSEI) in order to study medical colleges. Fanslow specifically developed the College Environment Inventory for Women (CEIW) for a study on female college students [19, 20]. Furthermore, other tools include the Classroom Environment Scales (CES), the Inventory of College Characteristics (ICCS), the Learning Environment Inventory (LEI), the College and University Environment Inventory (CUCEI), the Institutional Goals Index (IGI), and the Institutional Functioning Inventory (IFI) [21].

Recently, the Dundee Ready Education Environment Measure (DREEM) has been widely used internationally to evaluate students' perceptions regarding the various aspects of educational environments [22, 23]. Dr. Sue Roff of the University of Dundee in England developed DREEM in 1997 using the Delphi method [21]. Its questionnaire items were translated and used in at least 20 countries [22]. Its validity and reliability were verified [21, 22]. But some studies indicated poor data on the five-factor structure and construct validity. They recommended the need for further research and improvement [24–36].

In Korea, a DREEM-based survey was distributed across 40 medical colleges nationwide in 2013 [37] and in one college of Korean medicine in 2015 [38]. Because the DREEM was originally developed in English, this international tool includes some problems associated with language issues and multicultural populations [39, 40]. To avoid the ambiguities and inconsistencies with regard to meaning on word in each version, a strict translation process should be applied for considering the equivalence between the original language version and the target language version. Cross-cultural adaptation is a normative methodology used by researchers in order to consider the differences in languages and cultural backgrounds [41].

In this study, we conducted a series of cross-cultural adaptation processes to develop a Korean version of DREEM based on the English version. We also performed a psychometric study with confirmative factor analysis to identify whether the latent variable structure of the original version could be applied to Korean medicine students.

## 2. Methods

### 2.1. The Dundee Ready Educational Environment Measure Questionnaire

DREEM has fifty items and a 5-factor structure: SPL (students' perception of learning [12 items]), SPT (students' perception of teachers [11 items]), SAS (students' academic self-perception [8 items]), SPA (students' perception of atmosphere [12 items]), and SSS (students' social self-perception [7 items]).

A bipolar 5-point Likert scale with a neutral response is applied (0: strongly disagree; 1: disagree; 2: neither agree nor disagree; 3: agree; 4: strongly agree) to the 50 items. Among the fifty items, nine items (item 4, 8, 9, 17, 25, 35, 39, 48, and 50) are negative sentences; therefore, they should be reversely coded when scoring and analysis are performed. High scores indicate a positive evaluation regarding the education environment. The developers provided an interpretation guideline for each item or each factor. According to that guideline, items or factors with a score equal to or greater than 3.5 indicate a very positively evaluated area; items or factors with a score between 2 and 3 indicate a positively evaluated area but, however, require some efforts to enhance education environment, and items or factors with a score equal to or less than 2.0 indicate that have to demand attention to education environment [42]. The DREEM questionnaire enables researchers to quantitatively assess students' perceptions of the educational environment. It also makes it feasible to compare results across different institutions, various curriculums, and different survey researches [17].

### 2.2. Cross-Cultural Adaptation Process

Wherever the cross-cultural adaptation guideline is presented, there is a slight difference in process; however, in general, a serial process of translation, back translation, review, and pretesting is performed [39–41, 43–45]. The World Health Organization (WHO) guidelines also recommend a serial process of forward translation, expert panel back translation, pretesting, cognitive interviewing, and fixing the final version [46]. In this study, we referred to the adaptation guidelines written by Beaton [39] as well as Sousa and Rojjanasrirat's [40] six stages.

**Figure 1:**
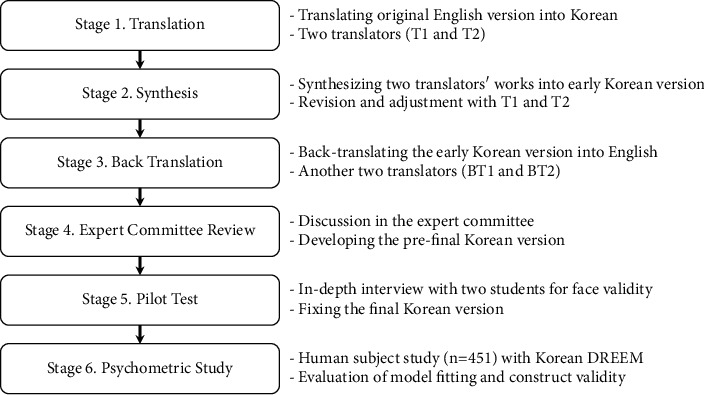
The cross-cultural adaptation process of this study.

Before starting this study, the authors informed Dr. Sue Roff, the original developer of DREEM, about this cross-cultural adaptation and received her permission along with her kind and helpful advice via e-mail.  Figure 1 depicts the overall process underlying the cross-cultural adaptation of DREEM.

During the first stage, two translators independently translated the English DREEM items into Korean. Their mother tongue was Korean, but they could speak English fluently. We provided this research's aims and advance information to translator 1 (T1), while translator 2 (T2) was provided with no information regarding this study. During stage 2, we synthesized the two products obtained from T1 and T2. After we showed the synthesized version to T1 and T2, we received feedback from the two translators and revised the manuscript (early Korean version). During stage 3, two other translators independently translated the early Korean version produced in stage 2 into English. The two back-translators (BT1 and BT2) were not provided with any information on the original version of DREEM and the medical education system. During stage 4, we organized an expert committee where we developed the prefinal Korean version of DREEM after considering and analyzing all the products obtained from T1, T2, BT1, and BT2. A total of eight experts participated in the committee: one survey research methodologist, one healthcare professional, one language professional, one education professional, and four translators (T1, T2, BT1, and BT2). The first two members of the committee, and two translators (T1 and T2), were Korean medicine doctors who worked in the college of Korean medicine. Throughout stage 4, the committee members focused on semantic equivalence, idiomatic equivalence, experiential equivalence, and conceptual equivalence in order to maintain the equivalence between the original English version and the new Korean version [39, 43]. Dr. Sue Roff's answers and advice were very helpful during this stage. We were able to develop the prefinal Korean version of DREEM in stage 4. During the next stage, we conducted an in-depth interview with two students to confirm the clarity of the items. They provided advice from a student's point of view, and almost no changes were made in prefinal version. Consequently, the Korean version of DREEM was finalized. During the final stage, we performed a psychometric study using the Korean version of DREEM. This was followed by statistical analysis including descriptive study and confirmatory factor analysis.

### 2.3. Psychometric Testing and Statistical Analysis

After completing the cross-cultural adaptation, we performed a human subject research using the newly developed Korean version of DREEM. A survey was administered to second-year to sixth-year students in two universities. The online survey platform Survey Monkey (Survey Monkey, CA, USA) was used for collecting the responses. A total of 218 students and 233 students from two universities, respectively, voluntarily participated in the online survey. This human subject research was approved by the Institutional Review Board of Kyung Hee University Korean Medicine Hospital (KOMCIRB-161020-HR-059).

We performed descriptive statistical methods in order to summarize the basic characteristics and responses to the survey and confirmatory factor analysis to compare the latent variable structure of this population with the original structure and previous studies. For evaluating the goodness of fit of the original model using the acquired dataset, several model fit indices were calculated and discussed: goodness-of-fit index (GFI), adjusted goodness-of-fit index (AGFI), root mean square error of approximation (RMSEA), normed fit index (NFI), Tucker–Lewis index (TLI), and comparative fit index (CFI). With these model fit indices, we investigated the factor loading of each item, the construct reliability (CR) for the convergent validity, and the average variance extracted (AVE) for the discriminant validity. We used Microsoft Excel Office 365 (Microsoft, Redmond, WA, USA) for performing the descriptive statistics and R 3.6.1. (R Core Team, Vienna, Austria) and R packages “sem” for performing confirmatory factor analysis.

## 3. Results

### 3.1. Cross-Cultural Adaptation of the DREEM Questionnaire

Table 1 presents the Korean version of DREEM, which was developed using the cross-cultural adaptation process.

### 3.2. Psychometric Test Results

#### 3.2.1. Demographic Statistics and the Overall and Subgroup Analysis of the Psychometric Test

A total of 451 students from two Korean medicine colleges voluntarily responded to the survey inquiry. All items were answered, and there was no missing data. Tables 2 and 3 present the overall results and subgroup analysis results, respectively. The descriptive statistics provided in the tables show that over half of the items were identified as being problematic, with scores equal to or less than 2.0. The students responded negatively to many items in general. Almost no differences were found between colleges and genders and among school years.

#### 3.2.2. Confirmatory Factor Analysis of the Psychometric Test

**Figure 2:**
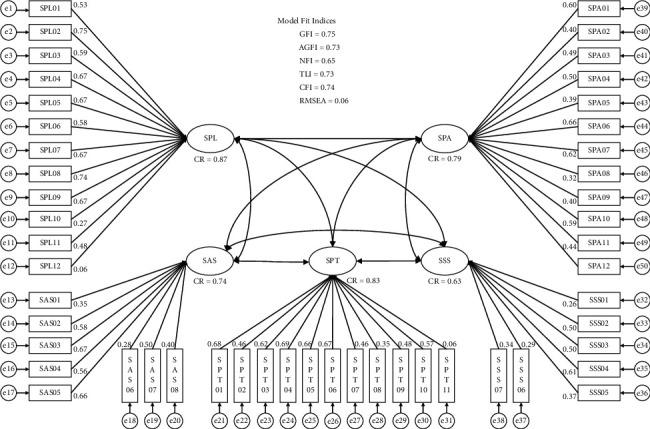
The confirmatory factor analysis model and the model fit indices.

Figure 2 presents the result of the confirmatory factor analysis of the 5-factor model based on the original DREEM questionnaire with the acquired dataset. Factor loadings and construct reliabilities (CRs) for evaluating the convergent validity are also presented in the same figure. The indices for model fit are as follows: GFI = 0.75, AGFI = 0.73, NFI = 0.65, TLI = 0.73, CFI = 0.74, and RMSEA = 0.06.  Table 4 shows the correlation coefficients and the average variance extracted (AVE) for evaluating the discriminant validity.

## 4. Discussion

### 4.1. The Cross-Cultural Adaptation Process

Cultural and linguistic diversity has caused problems with regard to translation, and many researchers have continued to study translation methods [47]. As a result, a general consensus has been formed for the cross-cultural adaptation process.

Numerous multinational studies have contributed toward DREEM's development. It has been translated into various languages, used in various countries for many years, and has been identified as a nonculturally specific questionnaire [21, 22]. Some studies differed from consensus of cross-cultural adaptation process, such as multiple translators not participating, missing the back translation, or proceeding in a different order [26, 48–51]. Some did not elaborate on whether they followed the consensus process or not [15, 24, 37, 52–56].

This study encountered some difficulties because the expert committee found some expressions to be unclear during the process of creating the prefinal version. In response, the final version was produced after a comparison with other questions, agreements between experts, and communication with the original writer (S. Roff). In the cases of item #12 “timetable,” item #31 “empathy in my profession,” and item #33 “in class socially,” it was necessary to accurately define the meaning of the words. “Timetable” can be interpreted in various ways including a syllabus or a class schedule. Roff's response was that it included all such meanings. The meaning of “my professional” in item #31 also included both doctor-doctor (the ability to form consensuses between doctors, the ability to discuss diagnoses, etc.) and patient-doctor relationships. In the case of item #33, “class” indicated all courses of study, including lectures and seminars. Internationally, the medical curriculum is not unified, and this can result in differences in terminology. The term “ward teaching” is translated into the Korean term “hoe-jin,” which refers to clinical practice conducted by professors, which involves seeing and examining patients in the ward. In Korea, ward teaching is usually conducted in the 6^th^ year of Korean medicine college. In the case of “constructive criticism” in item #32, students may find the term “constructive” vague. Based on the words and examples provided by Roff, a description that could supplement the word was added in the Korean version. In the case of item #34, we needed to grasp the meaning of individual “seminars/tutorials.” Roff suggested that the word was basically referred to a small number of people and that it was a concept that contrasted with the lectures. Therefore, “seminars/tutorials” was replaced by small group classes/small group activities in individual practice sessions.

### 4.2. Basic Statistics and the Evaluation of Construct Validity

The descriptive statistics of the results showed that many students were not satisfied with the education environments in Korean medicine colleges. The total and subscores of DREEM were almost equal to or less than half score, and 58% of items were identified as being problematic. This result is similar to that of a previous study in Korea [38]. Thus, education policy on Korean medicine requires further effort and investment.

Next, we examined whether the original 5-factor model of DREEM was suitable for the new dataset obtained from Korean medicine students. We conducted confirmatory factor analysis and calculated several parameters, including goodness-of-fit indices, convergent validity, and discriminant validity.

First, the goodness of fit can be evaluated when the original 5-factor model is applied to the new dataset. Generally, it has been known that RMSEA less than 0.05 is considered to be good, RMSEA between 0.05 and 0.08 is acceptable, RMSEA between 0.08 and 0.1 is marginal, and RMSEA over 0.1 is poor [57]. The RMSEA for our study was 0.06. We concluded that the original 5-factor model was acceptable for the new dataset obtained from the Korean medicine students. However, the other indices for model fit—GFI, AGFI, NFI, TLI, and CFI—were around 0.7. Among them, TLI and CFI, which were relatively sample size independent, were 0.73 and 0.74. In other countries, they also had results of about 0.7 [30, 32, 35], respectively, although it is suggested that these indices should be over 0.9 for a good model fit [58, 59]. Based on these results, we concluded that the goodness of fit of this model with our dataset was acceptable but not very good. Such less satisfactory results can imply different concept structures between countries or cultures.

Second, the convergent validity was examined using factor loadings and CRs. Ideally, they must be equal to or greater than 0.707 to demonstrate desirable convergent validity [60–62], but values over 0.5 are also generally accepted [63]. Among 50 items, only 2 items showed factor loading values over 0.707, and 28 items showed factor loading values over 0.5; however, the CRs of each factor (except for latent variable SSS) were all over 0.707. Based on these indices, we can consider the relatively low convergent validity of the model in this study. Low convergent validity indicates that the items cannot be explained by only the corresponding latent variable and that some other factors are influencing the information of the items. To consider good discriminant validity, the AVE of one factor should be larger than the shared variance estimates with any other factor including measurement errors [64, 65]. In this study, except for the only SPT-SSS pair, all squares of correlation coefficients were greater than the corresponding AVEs. Low convergent validity and low discriminant validity indicated that the original 5-factor model was not suitable for our dataset, which was obtained from Korean medicine students.

This result may have two possible explanations. First, as shown in the basic statistics, many respondents gave low scores across all the items. Such overall low scores may make it difficult to separate the latent variables, and this may induce low construct validity. Second, because of the differences in languages and cultures, the Korean students' perceptions regarding medical education environments may have different structures from the perception of the other countries' students. In order to clarify this issue, further studies with large amounts of samples should be conducted.

### 4.3. Limitations and Further Research

This study aimed to develop a Korean version of DREEM to promote more active use of DREEM in Korea and to reduce errors in the comparisons of Korean study results and those of other countries. Moreover, we aimed to obtain methodological justification through a strict process of cross-cultural adaptation, not simple translation by a few bilingual translators. Because of differences in terms of languages, cultures, medical systems, and education systems, the meanings of some items had to be modified despite the kind and considerable help of the original developer. We consider this to be an unavoidable aspect of localization that occurs in cross-cultural adaptation.

With regard to statistics, the convergence in each factor and the discrimination among factors were not clear. Therefore, our data were not considered to be suitable for the original 5-factor model. As has been suggested by some previous studies, five-factor structure of DREEM (original DREEM subscales) was not good model fit and new structure or abridged version was suggested [24–36]. Through further additional human subject researches in Korea, the structure model can be modified or respecified using consecutive confirmatory factor analysis or exploratory factor analysis.

Therefore, if researches using the Korean version of DREEM, which has undergone cross-cultural translation in this study, are conducted continuously, it could be possible to make policy decisions by analyzing what resource aspects should be put on to improve the quality of medical educational environments through comparison studies between educational institutions and countries. Moreover, it could be possible to objectively evaluate the effect of used resources and improved education systems if data are collected in time series for a specific population.

## 5. Conclusion

In conclusion, we developed the Korean version of DREEM using the guidelines of cross-cultural adaption in order to create an objective evaluation tool for evaluating medical education in Korea. The adaptation process encountered some difficulties; however, we succeeded in finalizing the final Korean version of DREEM with the original author's help and advice. Furthermore, we performed a human subject research and statistical analysis to confirm the construct validity of the translated version. Statistical analysis regarding validity showed that the original 5-factor structure had a somewhat acceptable fit. Nevertheless, with regard to aspects of convergent validity and discriminant validity, low validity indices suggested that further researches should be conducted in Korea in order to study respecified or modified factor structure. If this study's product is used widely in Korea, we expect that the medical educational environment could be improved by identifying and studying the distinct features of the medical education system in Korea.

## Figures and Tables

**Table 1:** The final Korean version of DREEM developed in this study.

Item	Subscale	Original version (Sue Roff, 1997)	Korean version (this study)
1	SPL	I am encouraged to participate in class	수업시간에 적극적으로 참여하도록 독려 받는다
2	SPT	The teachers are knowledgeable	교수들은 풍부한 지식을 갖고 있다
3	SSS	There is a good support system for students who get stressed	스트레스를 받는 학생들을 위한 지원체계가 잘 갖춰져 있다
4	SSS	I am too tired to enjoy this course	나는 너무 피로해서 학교생활을 즐기기 어렵다
5	SAS	Learning strategies which worked for me before continue to work for me now	이전에 효과가 있었던 공부법이 현재에도 효과가 있다
6	SPT	The teachers are patient with patients	교수들이 환자 중심적으로 접근하는 편이다
7	SPL	The teaching is often stimulating	수업은 흥미를 불러일으켜 주는 편이다
8	SPT	The teachers ridicule the students	교수들이 학생들을 비하한다
9	SPT	The teachers are authoritarian	교수들이 권위적이다
10	SAS	I am confident about passing this year	나는 올해 진급할 것을 확신한다
11	SPA	The atmosphere is relaxed during the ward teaching	병원실습 분위기는 편안하다
12	SPA	This school is well timetabled	우리 학교의 학사일정, 교육과정, 시간표는 잘 짜여 있다
13	SPL	The teaching is student centred	수업은 학생 중심적이다
14	SSS	I am rarely bored on this course	나는 학교생활이 거의 지루하지 않다
15	SSS	I have good friends in this school	나는 학교에 좋은 친구들이 있다
16	SPL	The teaching is sufficiently concerned to develop my competence	수업은 나의 역량을 계발하는데 도움이 된다
17	SPA	Cheating is a problem in this school	수업이나 시험에서 부정행위가 문제되고 있다
18	SPT	The teachers have good communications skills with patients	교수들은 환자들과 의사소통을 잘 하는 편이다
19	SSS	My social life is good	학교 내에서의 대인관계는 원만하다
20	SPL	The teaching is well focused	수업의 요점이 명확하다
21	SAS	I feel I am being well prepared for my profession	나는 의료인으로서의 전문성이 잘 준비되고 있다고 느낀다
22	SPL	The teaching is sufficiently concerned to develop my confidence	수업은 자신감을 키우는데 도움이 된다
23	SPA	The atmosphere is relaxed during seminars/tutorials	강의식 수업 분위기는 편안하다
24	SPL	The teaching time is put to good use	수업시간은 잘 활용된다
25	SPL	The teaching overemphasizes factual learning	수업이 지식 전달에 치우쳐 있다
26	SAS	Last year's work has been a good preparation for this year's work	작년에 공부한 것들이 올해 공부하는데 큰 도움이 되었다
27	SAS	I am able to memorize all I need	나는 필요한 모든 것을 암기 할 수 있다
28	SSS	I seldom feel lonely	나는 거의 외롭지 않다
29	SPT	The teachers are good at providing feedback to students	교수들은 학생들에게 적절한 피드백을 준다
30	SPA	There are opportunities for me to develop interpersonal skills	대인관계능력을 계발할 수 있는 기회가 있다
31	SAS	I have learned a lot about empathy in my profession	의료인으로서의 공감능력을 많이 습득해 왔다
32	SPT	The teachers provide constructive criticism here	교수들은 발전적인 방향으로 격려한다
33	SPA	I want feel comfortable in class socially	나는 수업구성원들 간의 관계에서 편안함을 느낀다
34	SPA	The atmosphere is relaxed during seminars/tutorials	소그룹수업/조별활동의 분위기가 편안하다
35	SPA	I find the experience disappointing	학교를 다니면서 실망스러운 경험을 하곤 한다
36	SPA	I am able to concentrate well	나는 집중을 잘 할 수 있다
37	SPT	The teachers give clear examples	교수들은 명확한 예시를 활용한다
38	SPL	I am clear about the learning objectives of the course	나는 각 수업의 학습목표들을 명확히 이해한다
39	SPT	The teachers get angry in class	교수들이 수업 중에 화를 낸다
40	SPT	The teachers are well prepared for their classes	교수들은 수업준비를 잘 한다
41	SAS	My problem-solving skills are being well developed here	나의 문제 해결 능력이 향상되고 있다
42	SPA	The enjoyment outweighs the stress of studying medicine	학업을 통해 얻는 즐거움이 학업 스트레스보다 크다
43	SPA	The atmosphere motivates me as a learner	학교 분위기가 학습의욕을 향상시킨다
44	SPL	The teaching encourages me to be an active learner	수업을 통해 능동적으로 학습하도록 독려 받는다
45	SAS	Much of what I have to learn seems relevant to a career in medicine	학습내용 대부분은 졸업 후 직무와 관련이 있는 편이다
46	SSS	My accommodation is pleasant	학생 편의시설은 쾌적하다
47	SPL	Long-term learning is emphasized over short term	장기학습을 단기학습보다 강조하고 있다
48	SPL	The teaching is too teacher-centred	수업이 지나치게 교수 중심적이다
49	SPA	I feel able to ask questions I want	내가 필요한 것을 편안하게 물어볼 수 있다
50	SPT	The students irritate the teachers	학생들이 교수들을 짜증나게 한다

**Table 2:** Overall results of the psychometric test (*N* = 451).

Subscale of DREEM (number of items)	Max	Min	Mean	SD	Number of problematic items
SPL (12)	40	2	20.08	6.76	10
SPT (11)	39	2	22.12	5.97	4
SAS (8)	32	1	16.74	4.59	3
SPA (12)	44	3	21.53	6.03	8
SSS (7)	24	1	13.60	3.48	4
Total (50)	170	27	94.06	22.76	29

All data were acquired using a 5-point Likert scale (0–4). DREEM: Dundee Ready Educational Environment Measure. SD: standard deviation. SPL: students' perception of learning; SPT: students' perception of teachers; SAS: students' academic self-perception; SPA: students' perception of atmosphere; SSS: students' social self-perception.

**Table 3:** Subgroup results of the psychometric test (*N* = 451).

	College		Gender		School years				
Subscale (perfect score)	A	B	Male	Female	2^nd^	3^rd^	4^th^	5^th^	6^th^
	*N* = 218	*N* = 233	*N* = 237	*N* = 214	*N* = 86	*N* = 81	*N* = 93	*N* = 90	*N* = 101
SPL (48)	20.00 (7.09)	20.14 (6.44)	19.26 (6.99)	20.98 (6.39)	22.43 (6.47)	20.38 (6.82)	20.47 (6.68)	20.10 (6.62	17.44 (6.38)
SPT (44)	22.78 (6.07)	21.50 (5.81)	21.34 (6.10)	22.99 (5.71)	24.10 (5.24)	21.09 (6.27)	22.65 (5.81)	2.41 (5.86)	20.52 (6.06)
SAS (32)	16.88 (4.42)	16.61 (4.74)	16.41 (4.86)	17.11 (4.24)	17.65 (4.29)	16.09 (5.35)	16.63 (4.25)	17.49 (4.17)	15.91 (4.66)
SPA (48)	21.58 (6.28)	21.48 (5.80)	21.13 (5.87)	21.97 (6.19)	23.60 (5.58)	21.26 (6.55)	21.43 (5.97)	21.46 (6.09)	20.13 (5.61)
SSS (28)	13.54 (3.56)	13.66 (3.42)	13.46 (3.43)	13.76 (3.54)	14.37 (3.37)	13.30 (4.11)	13.18 (3.18)	13.43 (3.45)	13.72 (3.27)
Total (200)	94.79 (23.55)	93.39 (22.02)	91.60 (22.91)	96.79 (22.32)	102.2 (20.66)	92.11 (25.36)	94.37 (21.58)	94.89 (22.26)	87.72 (21.99)

All data were acquired using a 5-point Likert scale (0–4) and expressed as mean values (standard deviation). DREEM: Dundee Ready Educational Environment Measure. N: number of respondents. SPL: students' perception of learning; SPT: students' perception of teachers; SAS: students' academic self-perception; SPA: students' perception of atmosphere; SSS: students' social self-perception.

**Table 4:** Correlation coefficients, average variances extracted (AVEs), and construct reliabilities (CRs) of subscales for the convergent validity and the discriminant validity.

	SPL	SPT	SAS	SPA	SSS	AVE	CR
SPL	1					0.55	0.87
SPT	0.87 (0.76)	1				0.54	0.83
SAS	0.92 (0.85)	0.75 (0.56)	1			0.54	0.74
SPA	0.97 (0.94)	0.83 (0.69)	0.92 (0.85)	1		0.48	0.79
SSS	0.82 (0.67)	0.67 (0.45)	0.80 (0.64)	0.98 (0.96)	1	0.5	0.63

The squares of correlation coefficients are expressed in parentheses. SPL: students' perception of learning; SPT: students' perception of teachers; SAS: students' academic self-perception; SPA: students' perception of atmosphere; SSS: students' social self-perception; AVE: average variance extracted; CR: construct reliability.

## Data Availability

The datasets used and/or analyzed during the current study are available from the corresponding author on reasonable request.
